# Comparison of Mean and Centroid of Surgically Induced Astigmatism After Standard Cataract Surgery

**DOI:** 10.3389/fmed.2021.670337

**Published:** 2021-06-04

**Authors:** Kazutaka Kamiya, Kei Iijima, Wakako Ando, Nobuyuki Shoji

**Affiliations:** ^1^Visual Physiology, School of Allied Health Sciences, Kitasato University, Kanagawa, Japan; ^2^Department of Ophthalmology, School of Medicine, Kitasato University, Kanagawa, Japan

**Keywords:** surgically induced astigmatism, corneal astigmatism, mean, centroid, vector analysis, temporal incision, clear corneal incision, cataract surgery

## Abstract

**Purpose:** To compare the arithmetic mean of surgically induced astigmatism (M-SIA) and the centroid of surgically induced astigmatism (C-SIA) after standard cataract surgery.

**Methods:** We retrospectively examined 200 eyes of 100 consecutive patients undergoing bilateral cataract surgery through a 2.8 mm temporal clear corneal incision. We quantitatively measured the magnitude and axis of corneal astigmatism preoperatively and 3 months postoperatively using an automated keratometer (TONOREFF-II, Nidek). We assessed the M-SIA, the C-SIA, and the double angle plots for the display of the individual SIA distributions.

**Results:** For bilateral data analysis, the magnitude of corneal astigmatism significantly increased from 0.66 ± 0.39 D preoperatively to 0.74 ± 0.46 D postoperatively (paired *t*-test, *p* = 0.012). The M-SIA was 0.50 ± 0.36 D. On the other hand, the C-SIA was 0.18 ± 0.60 D at an axis of 97°. For unilateral analysis, we obtained similar outcomes between the right and left eye groups.

**Conclusions:** According to our experience, standard cataract surgery induces the M-SIA by approximately 0.5 D. The magnitude of the C-SIA largely decreased to approximately 40% of the M-SIA, and the direction of the C-SIA showed a tendency toward with-the-rule astigmatism. It should be noted that the M-SIA was considerably different from the C-SIA, especially when selecting the appropriate toric IOL model and power.

## Background

Toric intraocular lens (IOL) implantation has been widely acknowledged as a safe and effective means for the treatment of cataract patients with corneal astigmatism (1–3). Although modern sophisticated cataract surgery does not largely induce astigmatism due to a 2.0 to 3.0 mm-incision size as well as the unnecessity for sutures to the wound, it is essential to accurately predict surgically induced astigmatism (SIA) to maximize visual function and subsequent patient satisfaction, especially in toric IOL-implanted eyes. The arithmetic mean (M-SIA) calculation is based on the magnitude of astigmatism, but does not include the direction of astigmatism. By contrast, the centroid of SIA (C-SIA) is determined by both the magnitude and the direction of astigmatism. Therefore, it is reasonable that the use of the C-SIA is beneficial to grasp the overall SIA trends of cataract surgery, and thus the C-SIA, instead of the M-SIA, should be theoretically applied in toric IOL calculation software and might be somewhat different from the M-SIA. To the best of our knowledge, a detailed comparison between the M-SIA and the C-SIA has not so far been conducted. Moreover, the bilateral differences in these two SIAs between the right and left eyes have not been fully understood. It may give us intrinsic insights into the current understanding of astigmatic correction, especially when using a toric IOL model in daily practice.

The purpose of the present study is 2-fold; to retrospectively compare the M-SIA and the C-SIA, and to assess the bilateral differences in the two SIAs between the right and left eyes, after standard cataract surgery.

## Materials and Methods

### Study Population

The study protocol was registered with the University Hospital Medical Information Network Clinical Trial Registry (000043349). A total of 200 eyes of 100 consecutive patients (mean age ± standard deviation, 73.5 ± 8.1 years), who underwent bilateral standard phacoemulsification with IOL implantation through a 2.8 mm temporal clear corneal incision, and who completed a 3 month follow-up at Kitasato University Hospital, were enrolled in the current study. This retrospective review of the clinical charts was approved by the Institutional Review Board at Kitasato University Hospital (B20-302) and followed the tenets of the Declaration of Helsinki. Institutional review board approval was not required for this review.

### Inclusion and Exclusion Criteria

Inclusion criteria were 20 ≤ age < 95 years, eyes undergoing bilateral cataract surgery by one experienced surgeon, and no history of any trauma or ocular surgery. Exclusion criteria were eyes with concomitant corneal diseases, such as keratoconus, pellucid marginal degeneration, irregular corneal astigmatism, or severe dry eye, eyes developing intraoperative or postoperative complications, eyes requiring a wound enlargement, or eyes requiring some sutures to the wound. Written informed consent for cataract surgery was obtained from all patients.

### Cataract Surgical Procedures

Standard phacoemulsification was performed by capsulorhexis, nuclear and cortex extraction, and a non-toric IOL (KS-1, STAAR Japan, Chiba, Japan) implantation in the capsular bag through a 2.8 mm temporal clear corneal single-plane incision (4). All surgeries were performed by one experienced surgeon using the same technique. Postoperatively, steroidal (0.1% betamethasone), antibiotic (levofloxacin), diclofenac sodium medications were topically administered 4 times daily for 2 weeks, with the dose being steadily reduced thereafter.

### Assessment of Corneal Astigmatism and Surgically Induced Astigmatism

Preoperatively and 3 months postoperatively, we quantitatively assessed the magnitude and the axis of corneal astigmatism using an automated keratometer (TONOREFF-II, Nidek, Aichi, Japan). We measured this at least 5 times immediately after blinking according to the manufacturer's instructions and used the average value for statistical analysis. We assessed the M-SIA, the C-SIA by vector analysis, and the double angle plots for the display of the individual SIA distributions (5), by using the astigmatism double angle plot tool available on the American Society of Cataract and Refractive Surgery website (https://ascrs.org/tools/astigmatism-double-angle-plot-tool) (6).

### Statistical Analysis

The normality of all data samples was first checked using the Shapiro-Wilk test. Since the data fulfilled the criteria for normal distribution, the paired *t*-test was used for statistical analysis to compare the pre-and post-surgical data, as well as the right and left eye data. Unless otherwise indicated, the results are expressed as mean ± standard deviation, and a value of *p* < 0.05 was deemed statistically significant.

## Results

### Bilateral Data Comparison

**Figure 1:**
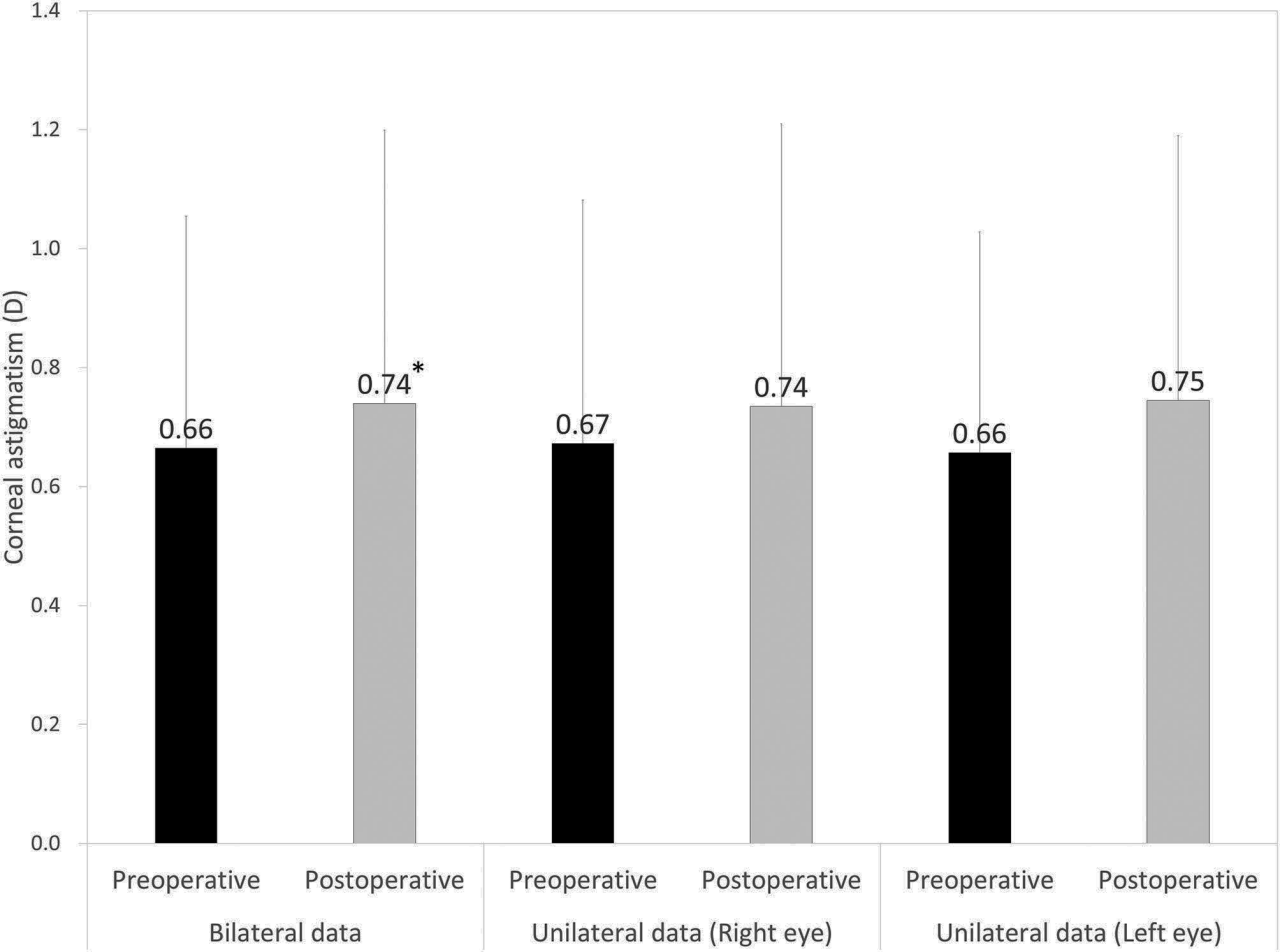
Graph showing the magnitude of corneal astigmatism preoperatively and 3 months postoperatively using bilateral and unilateral data. The bar represents standard deviation. D, diopters. *Indicates statistical significance.

**Figure 2:**
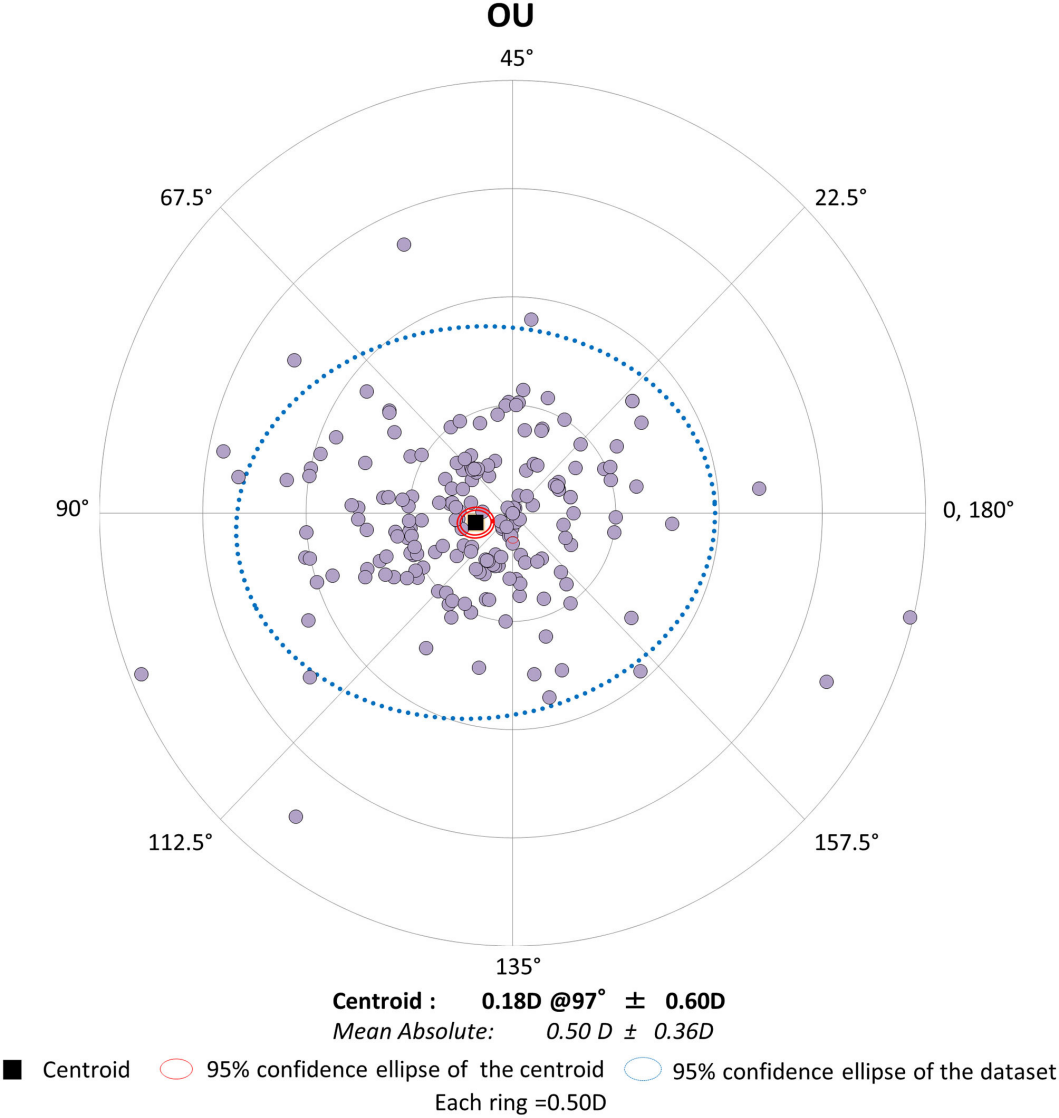
Graph showing the double angle plots of the individual surgically induced astigmatism using bilateral data.

Preoperative demographics of the study population are summarized in Table 1. We found no significant differences in the preoperative biometrics, such as uncorrected visual acuity (*p* = 0.509), or best spectacle-corrected visual acuity (*p* = 0.619), manifest sphere (*p* = 0.604), manifest cylinder (*p* = 0.710), corneal astigmatism (*p* = 0.751), mean keratometry (*p* = 0.599), central corneal thickness (*p* = 0.128), or axial length (*p* = 0.389), between the right and left eye groups. Visual and refractive outcomes are shown in Table 2. In the entire study population, the magnitude of corneal astigmatism significantly increased from 0.66 ± 0.39 D (range, 0.00 to 2.00 D) preoperatively to 0.74 ± 0.46 D (range, 0.00 to 2.25 D) postoperatively (paired *t*-test, *p* = 0.012) (Figure 1). The M-SIA was 0.50 ± 0.36 D (range, 0.00 to 2.17 D). On the other hand, the C-SIA was 0.18 ± 0.60 D at an axis of 97° (Figure 2).

**Table 1:** Preoperative demographic data of the study population through a 2.8 mm temporal clear corneal incision.

**Patient demographics**	
Age (years)	73.5 ± 8.1 years (range, 53 to 92 years)
Gender (M:F)	Male: 40, Female: 60
**Bilateral data**	
Flat keratometry (K1)	43.82 ± 1.44 D (range, 39.75 to 48.00 D)
Steep keratometry (K2)	44.49 ± 1.46 D (range, 40.50 to 48.25 D)
Mean keratometric readings (D)	44.16 ± 1.44 D (range, 40.13 to 48.13 D)
Corneal astigmatism (D)	0.66 ± 0.39 D (range, 0.00 to 2.00 D)
Axial length (mm)	23.76 ± 1.12 mm (range, 21.93 to 26.86 mm)
Central corneal thickness (μm)	548.6 ± 31.3 μm (range, 470 to 616 μm)
LogMAR UCVA	0.72 ± 0.42 (range, −0.08 to 2.00)
LogMAR BSCVA	0.26 ± 0.34 (range, −0.08 to 2.00)
Manifest sphere	−0.51 ± 3.10 D (range, −10.00 to 5.50 D)
Manifest cylinder	0.89 ± 0.82 D (range, 0.00 to 3.50 D)
**Unilateral data (right eye)**	
Flat keratometry (K1)	43.83 ± 1.42 D (range, 40.00 to 47.50 D)
Steep keratometry (K2)	44.50 ± 1.45 D (range, 40.50 to 47.75 D)
Mean keratometric readings (D)	44.16 ± 1.42 D (range, 40.25 to 47.63 D)
Corneal astigmatism (D)	0.67 ± 0.41 D (range, 0.00 to 2.00 D)
Axial length (mm)	23.77 ± 1.10 mm (range, 21.93 to 26.71 mm)
Central corneal thickness (μm)	547.9 ± 31.2 μm (range, 471 to 612 μm)
LogMAR UCVA	0.74 ± 0.44 (range, 0.05 to 2.00)
LogMAR BSCVA	0.27 ± 0.37 (range, −0.08 to 2.00)
Manifest sphere	−0.56 ± 3.16 D (range, −10.00 to 5.25 D)
Manifest cylinder	0.88 ± 0.77 D (range, 0.00 to 3.00 D)
**Unilateral data (left eye)**	
Flat keratometry (K1)	43.82 ± 1.47 D (range, 39.75 to 48.00 D)
Steep keratometry (K2)	44.48 ± 1.48 D (range, 40.50 to 48.25 D)
Mean keratometric readings (D)	44.15 ± 1.46 D (range, 40.13 to 48.13 D)
Corneal astigmatism (D)	0.66 ± 0.37 D (range, 0.00 to 1.50 D)
Axial length (mm)	23.75 ± 1.14 mm (range, 21.94 to 26.86 mm)
Central corneal thickness (μm)	549.3 ± 31.6 μm (range, 470 to 616 μm)
LogMAR UCVA	0.71 ± 0.40 (range, −0.08 to 1.70)
LogMAR BSCVA	0.25 ± 0.31 (range, −0.08 to 1.70)
Manifest sphere	−0.46 ± 3.06 D (range, −9.50 to 5.50 D)
Manifest cylinder	0.90 ± 0.88 D (range, 0.00 to 3.50 D)

**Table 2:** Visual and refractive outcomes of the study population undergoing standard cataract surgery through a 2.8 mm temporal clear corneal incision.

**Demographics**	**Preoperative**	**Postoperative**	***P*-value**
**Bilateral Data**			
LogMAR UCVA	0.72 ± 0.42 (range, −0.08 to 2.00)	0.23 ± 0.34 (range, −0.30 to 1.00)	<0.001
LogMAR BSCVA	0.26 ± 0.34 (range, −0.08 to 2.00)	−0.08 ± 0.08 (range, −0.30 to 0.00)	<0.001
Manifest sphere	−0.51 ± 3.10 D (range, −10.00 to 5.50 D)	−0.61 ± 1.05 D (range, −3.50 to 1.50 D)	0.572
Manifest cylinder	0.89 ± 0.82 D (range, 0.00 to 3.50 D)	0.41 ± 0.55 D (range, 0.00 to 2.00 D)	<0.001
**Unilateral Data (right eye)**			
LogMAR UCVA	0.74 ± 0.44 (range, 0.05 to 2.00)	0.24 ± 0.35 (range, −0.30 to 1.00)	<0.001
LogMAR BSCVA	0.27 ± 0.37 (range, −0.08 to 2.00)	−0.08 ± 0.08 (range, −0.30 to 0.00)	<0.001
Manifest sphere	−0.56 ± 3.16 D (range, −10.00 to 5.25 D)	−0.65± 1.03 D (range, −3.25 to 1.25 D)	0.705
Manifest cylinder	0.88 ± 0.77 D (range, 0.00 to 3.00 D)	0.38 ± 0.53 D (range, 0.00 to 2.00 D)	<0.001
**Unilateral Data (left eye)**			
LogMAR UCVA	0.71 ± 0.40 (range, −0.08 to 1.70)	0.23 ± 0.33 (range, −0.30 to 1.00)	<0.001
LogMAR BSCVA	0.25 ± 0.31 (range, −0.08 to 1.70)	−0.08 ± 0.08 (range, −0.30 to 0.00)	<0.001
Manifest sphere	−0.46 ± 3.06 D (range, −9.00 to 5.50 D)	−0.57 ± 1.08 D (range, −3.50 to 1.50 D)	0.675
Manifest cylinder	0.90 ± 0.88 D (range, 0.00 to 3.50 D)	0.45 ± 0.57 D (range, 0.00 to 2.00 D)	<0.001

*D, dioptre; LogMAR, logarithm of the minimal angle of resolution; UCVA, uncorrected visual acuity; BSCVA, best spectacle-corrected visual acuity*.

### Unilateral Data Comparison

**Figure 3:**
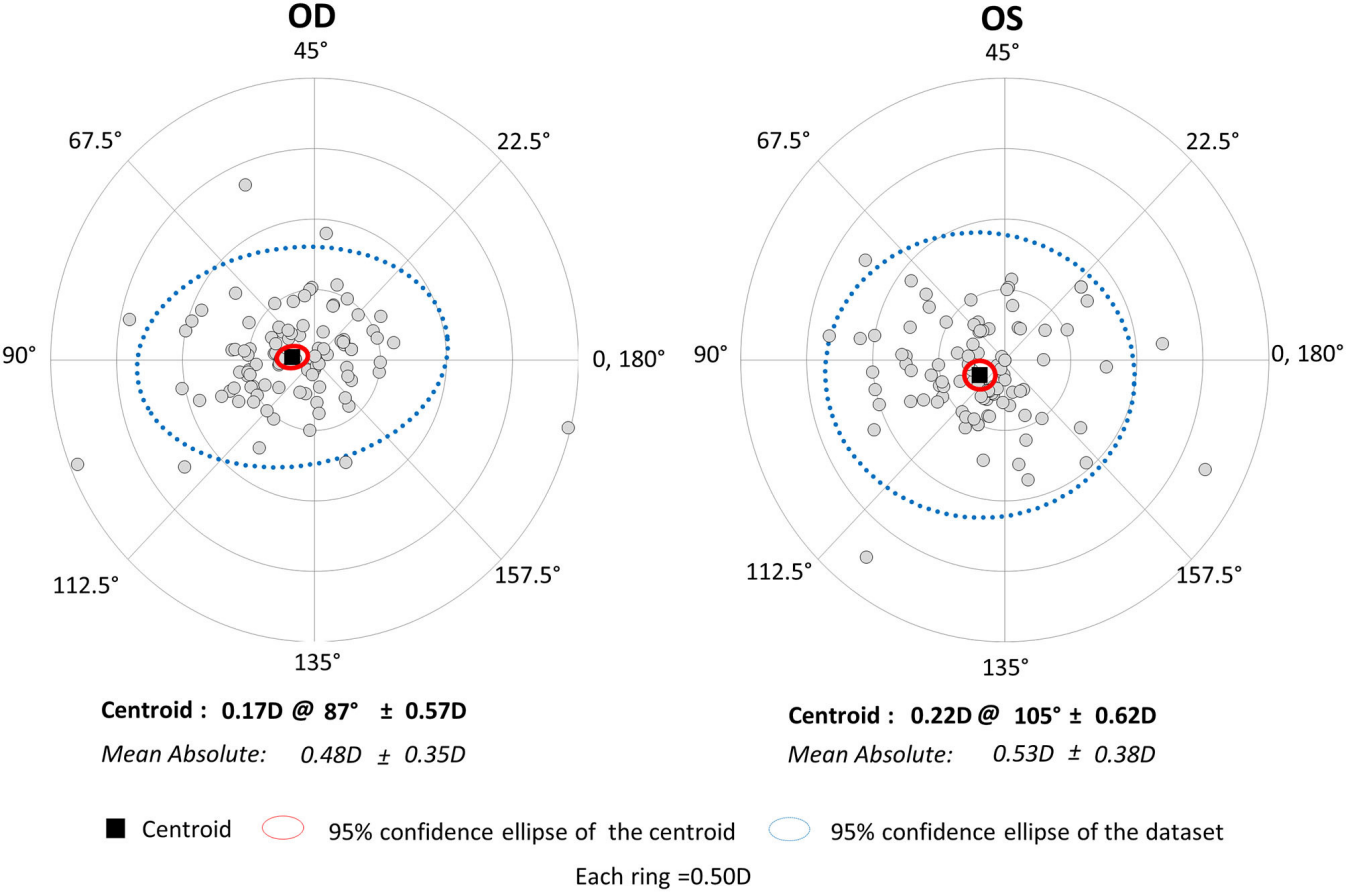
Graph showing the double angle plots of the individual surgically induced astigmatism using unilateral data.

The magnitude of corneal astigmatism did not significantly change from 0.67 ± 0.41 D (range, 0.00 to 2.00 D) to 0.74 ± 0.48 D (range, 0.00 to 2.25 D) (p = 0.111) in the right eye group. It did not significantly change from 0.66 ± 0.37 D (range, 0.00 to 1.50 D) to 0.75 ± 0.45 D (range, 0.00 to 2.00 D) (p = 0.056) in the left eye group (Figure 1). We found no significant differences in preoperative or postoperative astigmatism between the two groups (*p* = 0.751 or *p* = 0.851, respectively). The M-SIA was 0.48 ± 0.35 D (range, 0.02 to 1.98 D), and the C-SIA was 0.17 ± 0.57 D at an axis of 87° in the right eye group (Figure 3). The M-SIA was 0.53 ± 0.38 D (range, 0.00 to 2.17 D), and the C-SIA was 0.22 ± 0.62 D at an axis of 105° in the left eye group (Figure 3). We also found no significant differences in the M-SIA between the two groups (*p* = 0.248).

## Discussion

In the present study, our results showed that standard cataract surgery induced the M-SIA by ~0.5 D, and that the magnitude of corneal astigmatism similarly increased by ~0.1 D, even when using bilateral or unilateral data. Our results also showed that the magnitude of the C-SIA largely decreased to ~40% of the M-SIA. Interestingly, as shown in Figures 2, 3, the double angle plots of individual SIA showed various astigmatic vectors in magnitude and direction, and thus the C-SIA decreased to ~40% of the M-SIA in magnitude, and the direction of the C-SIA showed a tendency toward WTR astigmatism. We found no apparent differences in the M-SIA in magnitude or the C-SIA in magnitude or direction between the right and left eye groups. Although toric IOL model selection and IOL power calculation algorithm were not disclosed by the manufacturers, they were based on the astigmatism decomposition method. At present, some surgeons still prefer to select the appropriate toric IOL model and power by entering the M-SIA data, but not the C-MIA, in the manufacturer's software. Based on our present findings, we should be aware that the magnitude of the C-SIA was far smaller than that of the M-SIA, during preoperative planning for toric IOL implantation.

We assume that both SIAs were small in amount, but not necessarily negligible, since modern cataract surgery is deemed as one of the refractive surgeries that aims to correct both spherical and cylindrical errors as much as possible. We believe that this information may be simple, but clinically helpful, not only for cataract surgeons but also for IOL manufacturers, in order to further improve the astigmatic outcomes of toric IOL implantation.

Standard cataract surgery through a 3.0 mm temporal corneal incision has been reported to induce corneal astigmatism by ~0.5 D with a WTR shift in astigmatism (7–11). We also reported that phakic IOL implantation through the same 3.0 mm temporal incision induced similar astigmatism in magnitude and direction (12). These previous findings of the SIA were in good accordance with our current results of the M-SIA, but the C-SIA has not been meticulously investigated in their studies. We are of the opinion that the C-SIA is theoretically beneficial to grasp the overall SIA trends of cataract surgery, and thus that the C-SIA, instead of the M-SIA, should be applied for entering the SIA data in the manufacturer's toric IOL software during preoperative planning.

There are several limitations to this study. Firstly, we employed the autokeratometer for the assessment of corneal astigmatism, since it is still most widely utilized for astigmatic evaluation in daily practice. Accordingly, we did not evaluate posterior corneal astigmatism in this study. Although the amount of posterior corneal astigmatism is far smaller than that of anterior corneal astigmatism, total corneal astigmatism using a corneal tomographer would be beneficial for understanding the precise SIA of cataract surgery (13, 14). Secondly, we only examined the patients undergoing cataract surgery through a 2.8 mm clear corneal incision. We await further studies on the SIA using different incision sizes as well as different incisional techniques such as corneoscleral incision. Thirdly, we did not assess the effect of other contributing factors, such as corneal diameter, the type of the incision, and the intrastromal length of the incision on these SIAs in this study population. Theodoulidou et al. reported that a change >0.5 D of corneal astigmatism at 1 and 6 months postoperatively was significantly lower in eyes with a corneal diameter of 12.0 to 12.2 mm and ≥12.3 mm in comparison with eyes with a corneal diameter of ≤11.6 mm and 11.7 to 11.9 mm, indicating that the corneal diameter should always be measured preoperatively when planning cataract surgery and accounted for in cases of large and small corneas (15). On the other hand, Zhang et al. demonstrated that the horizontal corneal diameter had minimal effects on the SIA in uncomplicated small-incisional cataract surgery through a 2.2 mm clear corneal incision, indicating that the corneal diameter does not play a vital role in eyes undergoing small-incisional cataract surgery (16). Fourthly, we did not actually evaluate the astigmatic outcomes of toric IOL implantation. We are currently conducting a new comparative study on the astigmatic correction in toric IOL-implanted eyes by the use of the M-SIA and the C-SIA.

## Conclusions

In summary, our findings showed that standard cataract surgery through a 2.8 mm temporal corneal incision induced the M-SIA by ~0.5 D and that the magnitude of the C-SIA considerably decreased to ~40% of the M-SIA, with a WTR astigmatic shift in direction. Based on the fact that the M-SIA was largely different from the C-SIA in magnitude, the C-SIA, instead of the M-SIA, may be recommended to be applied, especially when we select the appropriate toric IOL model and power. This information will be helpful not only for cataract surgeons during preoperative planning, but also for IOL manufacturers, in order to further improve the astigmatic outcomes of toric IOL implantation.

## Data Availability Statement

The original contributions presented in the study are included in the article/supplementary material, further inquiries can be directed to the corresponding author/s.

## Ethics Statement

The studies involving human participants were reviewed and approved by the Institutional Review Board at Kitasato University Hospital (B20-302). Written informed consent for participation was not required for this study in accordance with the national legislation and the institutional requirements.

## Author Contributions

KK and NS were involved in the design and conducted the study. KI and WA were involved in the collection, management, analysis, and interpretation of data. KK, KI, WA, and NS were involved in preparation, review, and final approval of the manuscript. All authors contributed to the article and approved the submitted version.

## Conflict of Interest

The authors declare that the research was conducted in the absence of any commercial or financial relationships that could be construed as a potential conflict of interest.
